# Cardiomyopathy in a male patient with neutropenia and growth delay

**DOI:** 10.1186/1824-7288-40-45

**Published:** 2014-05-12

**Authors:** Veronica Folsi, Nunzia Miglietti, Annamaria Lombardi, Sara Boccacci, Tatiana Utyatnikova, Chiara Donati, Livia Squassabia, Laura Gazzola, Ilaria Bosio, Adele Borghi, Veronica Grassi, Lucia D Notarangelo, Alessandro Plebani

**Affiliations:** 1 Department of Pediatrics, Pediatrics Clinic, Spedali Civili of Brescia, Brescia, Italy; 2 Department of Pediatrics, Pediatric Cardiology Unit, Spedali Civili of Brescia, Brescia, Italy; 3 Department of Paediatrics, Pediatric Oncoematology Unit, Spedali Civili of Brescia, Brescia, Italy; 4 Pediatrics Clinic, Department of Clinical and Experimental Sciences, University of Brescia, Brescia, Italy

**Keywords:** Barth syndrome, Neutropenia, Cardiomyopathy, Growth delay, Tafazzin gene

## Abstract

Neutropenia encompasses a family of neutropenic disorders, both permanent and intermittent, ranging from severe (<500 neutrophils/mm^3^) to mild (500–1500 neutrophils/mm^3^), which may also affect other organ systems such as the pancreas, central nervous system, heart, muscle and skin. Neutropenia can lead to life-threatening pyogenic infections whose severity is roughly inversely proportional to the circulating neutrophil counts.

When neutropenia is detected, an attempt should be made to establish the etiology, and to distinguish acquired forms (the most frequent, including post viral neutropenia and autoimmune neutropenia) and congenital forms (rare disorders) that may be either isolated or part of a complex rare genetic disease. We report on a male patient initially diagnosed with isolated neutropenia who later turned out to be affected with Barth syndrome, a rare complex inherited disorder.

## Background

Neutropenia is a laboratory finding associated with variable severity ranging from benign transient neutropenia, such as that due to mild viral infection, to congenital neutropenia (CN). The latter is generally characterized by a bone marrow failure with developmental arrest of myelopoiesis at the promyelocyte/myelocytic stage, leading to an absolute neutrophil count (ANC) in the periphery below 500/mm^3^. Affected patients are particularly prone to severe infections. Molecular and genetic advances in the field of CN has not only allowed a better classification of this disorder, based on the underlying genetic defect, but has also shed light on the molecular mechanisms regulating both development and function of neutrophils [1]. From the clinical point of view, two major groups of CN can be distinguished: isolated CN without associated organ involvement, and CN associated with syndromic features [1]. Thus, it is important that, when neutropenia is detected, patients should be carefully investigated for associated signs and symptoms that may underlie a more complex and rare disorder. We report on a patient initially diagnosed with isolated neutropenia in whom a diagnosis of Barth syndrome (BTHS) was achieved later in life after the identification of cardiomyopathy (CMP) during an hospital admission for a respiratory infection.

## Case report

The patient was born from an uneventful pregnancy with normal perinatal conditions. The patient's mother reported one previous miscarriage during the first weeks of gestation. At birth, the patient's body measurements were at the lower percentile: weight was 2.870 g (10th percentile), length 49 cm (10th percentile) and head circumference 32 cm (<3rd percentile). No family history of CMP was reported. The patient's clinical history included a first admission at the age of 9 days for omphalitis. During this admission, borderline neutropenia was found (ANC: 1010/mm^3^). He was discharged with indications to perform periodical controls of neutrophil count. During these periodical controls, a severe neutropenia (WBC: 6140/mm^3^; ANC: 80/mm^3^) was detected at the age of 30 days requiring hospital admission. Bone marrow aspirate showed a maturational arrest of neutrophil precursors at the promyelocytic stage. The routine laboratory work up, including serology for viruses and bacteria and anti-neutrophil-specific antibodies, was negative. Genetic analysis of the ELANE and HAX1 genes, mutations which account for 60-70% of congenital neutropenia, did not reveal any alteration. Treatment with Granulocyte-Colony Stimulating Factor (G-CSF) (10 μg/kg/day) was undertaken due to severe neutropenia and withdrawn after stable normalization of ANC (1600/mm^3^). The patient was discharged with diagnosis of CN and was regularly followed at the outpatient clinic.

**Figure 1:**
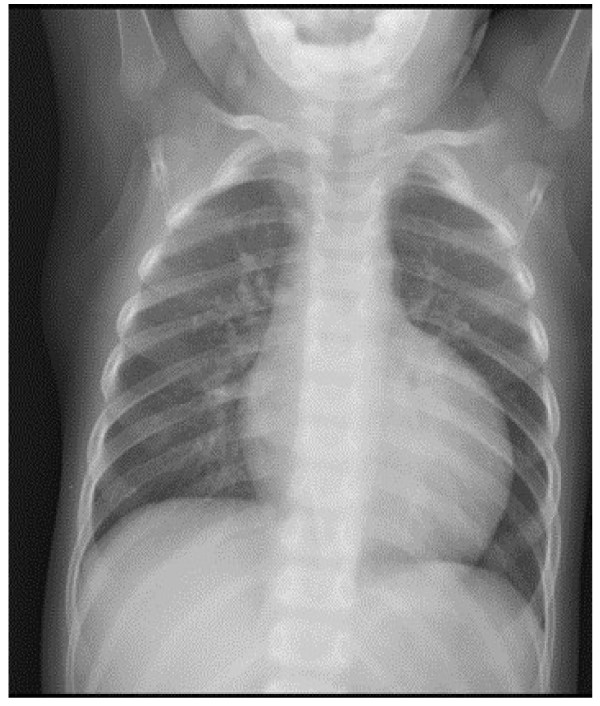
Chest X-ray showing severe cardiomegaly.

**Figure 2:**
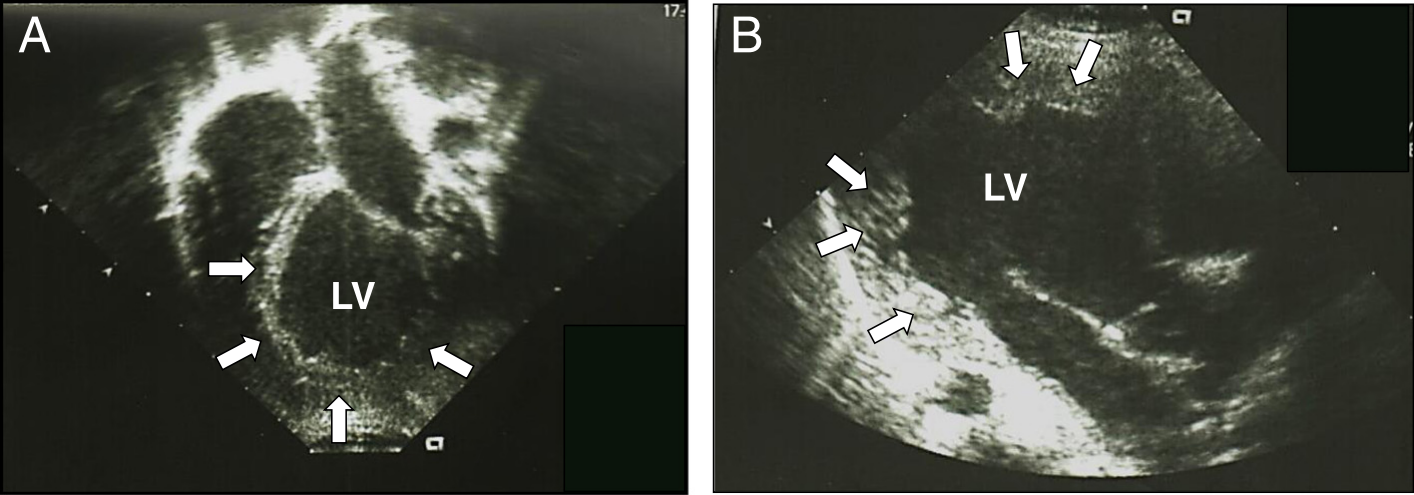
**Echocardiographic images of the index patient.** Both in apical four chamber **(A)** and in parasternal long axis view **(B)**. The left ventricle (LV) appears dilated and spherical in shape. Note the preserved thickness of the parietal and septal myocardium with the relative prominence of the trabeculae of the apex and lateral wall (arrows).

The patient was admitted again at the age of 24 months for febrile neutropenia (ANC: 400/mm^3^). His body measurements were still at the lower percentile for age: weight 10.4 Kg (3rd percentile) and height 82.5 cm (3rd percentile). Analysis of the growth charts confirmed the progressive growth delay. Auscultation revealed wheezing and crackles; pulse oximetry was 96%. Cardiac examination was unremarkable with a heart rate of 125 beats/minute and arterial pressure of 83/49 mmHg. Abdominal evaluation revealed hepatomegaly. Chest radiography ruled out pneumonia but revealed the presence of cardiomegaly (Figure 1). Echocardiographic evaluation documented dilated CMP, with marked enlargement of the left ventricle and severe impairment of the systolic function (left ventricular ejection fraction of 20%). Nevertheless left ventricular wall and septal thickness were preserved and myocardial trabeculature was unusually prominent, though not enough to fulfill the criteria for the diagnosis of left ventricular noncompaction (LVNC) [2] (Figure 2A and B). The pediatric cardiologist prescribed treatment with Furosemide 1 mg/Kg intravenously, Captopril 1 mg/kg and Spironolactone 2.5 mg/kg. No syndromic clinical features were evident and neurologic examination was unremarkable.

After reviewing the patient's features (neutropenia, growth delay, and cardiomyopathy), BTHS was taken into consideration. The presence of urine organic acids (elevated levels of urinary 3-methylglutaconic acid) strongly supported this diagnostic hypothesis. Genetic analysis of the tafazzin gene (*TAZ*), the causative gene of BTHS, revealed a c646 G > A substitution, leading to the aminoacidic substitution p.Gly216Arg, confirming therefore the diagnosis of BTHS. The patient was discharged in stable condition with the following maintainance therapy: Furosemide 0.5 mg/kg, Carvedilol 0.2 mg/kg, Enalapril 0.25 mg/kg and Aldactazide 1.5 mg/kg, with good cardiological control until the last outpatient visit. He was put on G-CSF treatment 3 μg/Kg three times a week, a dose which allowed to maintain ANC between 1500-2000/mm^3^.

## Discussion

Barth syndrome is a rare X-linked disorder caused by mutations in the *TAZ* gene, also named G4-5 gene, encoding for the tafazzin protein. This protein is a phospholipid transacylase which is located in the inner leaflet of the mitochondrial membrane and plays an important role in the remodeling of cardiolipin which is known to be critical in maintaining mitochondrial structure as well as being involved in mitochondrial apoptosis [3,4]. BTHS is the first known inborn error that affects cardiolipin. Although the classical clinical phenotype is characterized by CMP, often with LVNC morphology, neutropenia, skeletal myopathy, growth delay, and typical facial features, the presence of these findings can vary significantly, likely resulting in underdiagnosis of this disorder [3-5]. Furthermore, male fetal loss, stillbirth and severe neonatal illness or death have been found in multiple families with BTHS [6].

The molecular mechanisms leading to the variable expression of these clinical features are still largely unknown.

CMP is the major clinical feature in BTHS, with high prevalence in early life, which can present with different cardiac phenotypes [3,4,7,8]. LVNC is also commonly seen either alone or in conjunction with other CMP phenotypes and is characterized by trabeculations in left ventricle with associated wall motion abnormalities [9]. In our patient the atypical appearance of the apex and lateral wall of the left ventricle was observed, but the diagnostic criteria for LVNC were not entirely fulfilled [2]. About 70% of BTHS patients are recognized to have CMP in the first year and all those who developed CMP do so by 5 years of age [7]. However, a diagnostic delay for BTHS in patients with CMP has been reported. Data from Pediatric Cardiomyopathy registries of the USA suggest that 3-5% of all boys with CMP have BTHS [3]. Initial presentation of BTHS-associated CMP may mimic viral myocarditis or may become clinically evident during viral infections. BTHS should therefore always be included in the differential diagnosis of males presenting with CMP of apparent viral infection, especially when neutropenia is present (which could be ascribed to secondary bone marrow suppression by viral infection).

Neutropenia is very common in BTHS and may be persistent or intermittent [3]. However, there may be a wide variability of the neutrophil counts with some patients having extremely low levels of neutrophils and others having from mild decrease to normal levels of circulating neutrophils [7]. Therefore, the decision to undertake treatment with G-CSF is largely dependent on the patient’s clinical features and ANC [3]. Neutropenia may be the initial finding in an undiagnosed BTHS patient. Growth delay is another well-described clinical feature of BTHS [1,3,7,8].

In our patient, neutropenia, from mild to severe, was the first symptom of presentation early in life, of what was later diagnosed as BTHS. During follow up, apart from neutropenia, the only additional clinical finding was a persistent growth delay. Cardiomyopathy, apparently in the absence of clinical symptoms, was detected incidentally during an hospital admission at the age of 24 months. No other clinical features such as motor delay or skeletal myopathy became apparent by this age, thus, a clinical diagnosis was not made until the patient was diagnosed with dilated cardiomyopathy. However, an early diagnosis could have been suspected by linking chronic neutropenia to growth delay starting from the perinatal period.

In our case the finding of raised urinary levels of 3-methylglutaconic acid strongly supported BTHS diagnosis. However it is well known that this parameter is a rather non-specific indicator of the disease since cases have been reported where urinary 3-methylglutaconic acid levels have been normal in patients with TAZ mutations [3]. Recently a new method based on the CL4 and MLCL/CL4 (monolysocardiolipin/tetralinoleyl cardiolipin) analysis, has been proposed as a suitable highly sensitive diagnostic test for BTHS [10].

Previously, BTHS was often considered as a lethal disease during infancy and early childhood.

However, improvements in management of associated neutropenia and cardiac disease have resulted in better survival. Although mortality remains high during the first 4 years of life, patients are reported to survive into their late 40s [3,11] in response to improvements of supportive therapy. Thus clinicians should consider BTHS early in the course of the clinical evaluation of males with persistent idiopathic neutropenia since an early recognition of abnormal signs and symptoms associated with BTHS is crucial to improve the clinical outcome of the affected patients. Furthermore, genetic diagnosis of BTHS allows for a correct genetic counselling.

## Consent

Written informed consent was obtained from the patient’s parents for publication of this case report and any accompanying images. A copy of the written consent is available for review by the editor-in-chief of this journal.

## Abbreviations

BTHS: Barth syndrome; ANC: Absolute neutrophil count; CN: Congenital neutropenia; WBC: White blood cell; G-CSF: Granulocyte colony-stimulating factor; CMP: Cardiomyopathy; LVNC: Left ventricular noncompaction.

## Competing interests

The authors declare that they have no competing interests.

## Authors' contributions

NM, AL, SB, VG, LN (all attending pediatricians), AB (pediatric cardiologist) contributed to the diagnosis and provided clinical assistance; VF, TU, CD, LS, LG, IB, (all residents in Pediatrics) reviewed relevant articles on the literature, collected all the patient’s data and drew the first draft of the manuscript; AP contributed to the conception and design, and revisited critically the manuscript. All authors read and approved the final version.
